# Supplementary material for: Willingness to pay for cataract surgery and associated factors among cataract patients in Outreach Site, North West Ethiopia
Source: PLoS One. 2021 Mar 24;16(3):e0248618. doi: 10.1371/journal.pone.0248618 (PMC7990211; doi:10.1371/journal.pone.0248618)
Supplement: S1 File — (DOCX) [file pone.0248618.s001.docx]

| **Case Scenario for participants for Willingness to Pay for Cataract Surgery and Associated Factors among Cataract Patients in Outreach Site, North West Ethiopia**  **Case Scenario**  **Introduction:** Cataract is the clouding of the eye's natural lenses, and it is the leading cause of blindness worldwide. This problem can be caused by ageing, family history, hypertension, obesity, diabetes mellitus, smoking, significant alcohol consumption, and other courses. Cataract surgery is the only treatment option o solve this problem. Foreign donors fund this outreach program designed for cataract surgery. It aims to fight blindness due to cataracts by assuring the accessibility and equity of people living in districts. By now, you are getting these services free of charge because funding organizations cover the cost. However, in the future, this service will no longer be funded and sustainable  **Benefits of cataract surgery:**  Cataract surgery helps to improve vision, prevent avoidable blindness, increasing productivity, and improve quality of life.  As we have said before, to make the service sustainable and accessible, designing a cost-recovery model is very important.  After presenting the above scenario, the participants will be asked about their willingness to pay for cataract surgery with the below format. |
| --- |

| **Code** | **Questions** | **Options** | **Skip** |
| --- | --- | --- | --- |
| Q101 | Are you willing to pay for cataract surgery providing at outreach sites? | 1. Yes | Go to Q102 |
|  |  | 1. No | Dropped from the next biding game |
| Q102 | If the actual cost of cataract surgery for one eye at the Outreach Site is **1000 ETB**, are you willing to pay for it? | 1. Yes | Go to Q103 |
|  |  | 1. No | Go to Q105 |
| Q103 | If the operation's price for one eye at the Outreach Site is **1250 ETB**, are you willing to pay for it? | 1. Yes | Go to Q104 |
|  |  | 1. No | Go to Q107 |
| Q104 | If the operation's price for one eye at the Outreach Site is **1500 ETB**, are you willing to pay for it? | 1. Yes | Go to Q107 |
|  |  | 1. No | Go to Q107 |
| Q105 | If the operation's price for one eye at the outreach Site is **750 ETB**, are you willing to pay for it? | 1. Yes | Go to Q107 |
|  |  | 1. No | Go to Q106 |
| Q106 | If the price of the eye's operation at the outreach Site is **500 ETB**, are you willing to pay for it? | 1. Yes | Go to Q107 |
|  |  | 1. No | Go to Q107 |
| Q107 | What is the maximum price you are willing to pay for one eye's operation at the outreach site? | **__________**__________ETB | |

After presenting the above case scenario, all participants were asked:

***Are you willing to pay some positive price for cataract surgery?***

1. ***Yes***
2. ***No***

If yes, follow the below algorism





Iterative bidding technique elicits maximum willingness to pay for cataract surgery in outreach Sites, North West Ethiopia.

December 2018, Exchange rate: US$ 1=ETB 27.8.
