# Supplementary material for: Willingness to pay for cataract surgery and associated factors among cataract patients in Outreach Site, North West Ethiopia
Source: PLoS One. 2021 Mar 24;16(3):e0248618. doi: 10.1371/journal.pone.0248618 (PMC7990211; doi:10.1371/journal.pone.0248618)
Supplement: S1 Questionnaire — (DOCX) [file pone.0248618.s002.docx]

## S1 Questionnaire

Questionnaire No____________

| **Part 1: Socio-demographic characteristics** | | | |
| --- | --- | --- | --- |
| **Code** | **Questions** | **Options** | **Skip** |
| Q 101 | Sex | 1. Male 2. Female |  |
| Q102 | Age | ____________years |  |
| Q103 | Marital status | 1. Single 2. Married 3. Divorced 4. Widow |  |
| Q104 | Residence | 1. Urban 2. Rural |  |
| Q105 | Family size | ___________(Number) |  |
| Q106 | Household head | 1. Yes 2. No |  |
| Q107 | Education status | 1. Unable to read and write 2. Able to read and write 3. Primary school 4. Secondary school 5. College/university |  |
| Q108 | What is your occupation? | 1. Student 2. Farmer 3. Daily labor 4. Merchant 5. Government employed 6. Others(specify)_________ |  |

| **Part 2: Household Wealth characteristics** | | | | | | | | | | | | | | | | | |
| --- | --- | --- | --- | --- | --- | --- | --- | --- | --- | --- | --- | --- | --- | --- | --- | --- | --- |
| **Code** | | **Questions** | | | | **Options** | | | | | | | | **Skip** | | | |
| **Q201** | | What is the primary source of drinking water for the household? | | | | 1. Piped water in the dwelling 2. Piped water from public taps 3. dug water 4. Spring/river/ 5. bottled water 6. other ___________________ | | | | | | | |  | | | |
| **Q202** | | What type of fuel does your household mainly use for cooking? | | | | 1. Wood 2. Electricity 3. Gas 4. Charcoal 5. Animal dung 6. Other __________________ | | | | | | | |  | | | |
| **Q203** | | What kind of latrine does your family have? | | | | 1. Flush or pour-flush toilet 2. Ventilated improved pit latrine 3. Pit latrine with/without a slab 4. Public toilet 5. No facility/bush/field 6. Other specify_____________ | | | | | | | |  | | | |
| **Q204** | | From whom the house of the household is owned? | | | | 1. Private 2. Rent 3. Government 4. Relative 5. Other_______________ | | | | | | | |  |  |  |  |
| **Q205** | | What is the primary material of the roof? | | | | 1. Natural roof (Thatch/mud/no roof) 2. Rudimentary(plastic/wood/cardboard) 3. Finished (Corrugated iron/cement/ceramic) 4. Other (specify)_____________ | | | | | | | |  | | | |
| **Q206** | | What is the primary material on the floor? | | | | 1. Natural floor (dung/earth) 2. Rudimentary (wood planks/bambo) 3. Finished walls(cement/ceramic)   Other (specify)_____________ | | | | | | | |  | | | |
| **Q207** | | What is the primary material of the exterior wall of the house? | | | | 1. Natural walls (no walls/mud/cane) 2. Rudimentary walls (stone/wood with mud/) 3. Finished walls (wood/stone with cement) 4. Other (specify)_____________ | | | | | | | |  | | | |
| Q208 | | How many rooms are used by the household for sleeping only? | | | |  | | | | | | | |  | | | |
| Q209 | | **Does the household or any member of the household have:** | | | | **Yes** | | | | **No** | | | | **If yes, how many?** | | | |
|  | | 1. Radio? | | | |  | | | |  | | | |  | | | |
|  | | 1. Watch/clock? | | | |  | | | |  | | | |  | | | |
|  | | 1. Non-mobile phone? | | | |  | | | |  | | | |  | | | |
|  | | 1. Mobile phone? | | | |  | | | |  | | | |  | | | |
|  | | 1. Solar? | | | |  | | | |  | | | |  | | | |
|  | | 1. Television? | | | |  | | | |  | | | |  | | | |
|  | | 1. Refrigerator? | | | |  | | | |  | | | |  | | | |
|  | | 1. Computer? | | | |  | | | |  | | | |  | | | |
|  | | 1. An electric mitad? | | | |  | | | |  | | | |  | | | |
|  | | 1. An animal-drawn cart? | | | |  | | | |  | | | |  | | | |
|  | | 1. Beds with cotton? | | | |  | | | |  | | | |  | | | |
|  | | 1. Beds with a sponge? | | | |  | | | |  | | | |  | | | |
|  | | 1. Beds with spring? | | | |  | | | |  | | | |  | | | |
|  | | 1. Beds with bran/chaff? | | | |  | | | |  | | | |  | | | |
|  | | 1. Table? | | | |  | | | |  | | | |  | | | |
|  | | 1. Chair made of wood? | | | |  | | | |  | | | |  | | | |
|  | | 1. Chair (sofa)? | | | |  | | | |  | | | |  | | | |
|  | | 1. Bicycle? | | | |  | | | |  | | | |  | | | |
|  | | 1. Motorcycle? | | | |  | | | |  | | | |  | | | |
|  | | 1. Generator | | | |  | | | |  | | | |  | | | |
|  | | 1. Bajaj | | | |  | | | |  | | | |  | | | |
|  | | 1. Car or truck? | | | |  | | | |  | | | |  | | | |
|  | | 1. others______________ | | | |  | | | |  | | | |  | | | |
| **Q210** | | **Does your household own the following farming animals currently?** | | | | **Yes** | | | | **No** | | | | **If yes, how many?** | | | |
|  | | 1. Plough oxen | | | |  | | | |  | | | |  | | | |
|  | | 1. Fattened ox | | | |  | | | |  | | | |  | | | |
|  | | 1. Milk Cows | | | |  | | | |  | | | |  | | | |
|  | | 1. Heifer/ Bull/ Calf | | | |  | | | |  | | | |  | | | |
|  | | 1. Goats/ Sheep | | | |  | | | |  | | | |  | | | |
|  | | 1. Donkey /Mule/ Horse | | | |  | | | |  | | | |  | | | |
|  | | 1. Chicken | | | |  | | | |  | | | |  | | | |
|  | | 1. Beehive | | | |  | | | |  | | | |  | | | |
|  | | 1. Others | | | |  | | | | | | | |  | | | |
| **Q211** | | Do you have a separate kitchen? | | | | 1. Yes 2. No | | | | | | | |  | | | |
| **Q212** | | Do you have separate rooms for cattle? | | | | 1. Yes 2. No | | | | | | | |  | | | |
| **Q213** | | **Does the household have these fruit or crop productions produced in the last one year?** | | | | **Yes** | | | | **No** | | | | **If yes, how much the amount in quintals(100kg)** | | | |
|  | | 1. Teff | | | |  | | | |  | | | |  | | | |
|  | | 1. Barley | | | |  | | | |  | | | |  | | | |
|  | | 1. Wheat | | | |  | | | |  | | | |  | | | |
|  | | 1. Maize | | | |  | | | |  | | | |  | | | |
|  | | 1. Sorghum | | | |  | | | |  | | | |  | | | |
|  | | 1. Dagusa | | | |  | | | |  | | | |  | | | |
|  | | 1. Bean/ Pea /Chickpea /Lentil | | | |  | | | |  | | | |  | | | |
|  | | 1. Carrot | | | |  | | | |  | | | |  | | | |
|  | | 1. Red onion | | | |  | | | |  | | | |  | | | |
|  | | 1. White onion | | | |  | | | |  | | | |  | | | |
|  | | 1. Potatoes | | | |  | | | |  | | | |  | | | |
|  | | 1. Tomatoes | | | |  | | | |  | | | |  | | | |
|  | | 1. Others specify_____________________________ | | | | | | | | | | | |  | | | |
| Q214 | | Does any member of this household own any agricultural land? | | | | | | | 1. Yes 2. No | | | | | | | | |
| Q215 | | How many hectares of agricultural land do members of this household own? | | | | | | |  | | | | | | | | |
| Q216 | | Does any member of this household have a bank saving account? | | | | | | | ________________________ETB | | | | | | | | |
| **Part 3: Health-related variables** | | | | | | | | | | | | | | | |  |  |
| **Code** | | | **Questions** | **Options** | | | | | | | | **Skip** | | | |  |  |
| Q301 | | | Do you have families/friends who had a history of cataract surgery? | 1. Yes 2. No | | | | | | | |  | | | |  |  |
| Q302 | | | From where did you get the services? | 1. Government hospital 2. private hospital 3. campaign/outreach | | | | | | | | **If Yes for Q404** | | | |  |  |
| Q303 | | | Which eye is selected for surgery? | 1. Right 2. left 3. both | | | | | | | |  | | | |  |  |
| Q304 | | | For how long did you stay with the condition? | _________________ | | | | | | | |  | | | |  |  |
| Q305 | | | Did you seek eye care services for the cataract? | 1. yes 2. No | | | | | | | |  | | | |  |  |
| Q306 | | | Why did you not seek eye care services before? | ______________________ | | | | | | | | **If No for Q** | | | |  |  |
| Q307 | | | Is there any ocular comorbidity other than cataracts? | 1. Yes 2. No | | | | | | | |  | | | |  |  |
| Q308 | | | Is there any chronic systemic illness? | 1. Yes 2. No | | | | | | | |  | | | |  |  |
| Q309 | | | Do you have a health insurance scheme (Community based health insurance? | 1. Yes 2. No | | | | | | | |  | | | |  |  |
| **Part 4: Preoperative Visual function** | | | | | | | | | | | | | | | | |  |
| **Code** | **Questions** | | | | **Options** | | | | | | | | | | | |  |
|  | Because of your vision, much difficulty does you have, even with eyeglass, in_______________? If you do not perform the activity for reasons unrelated to your, mark checkbox “NA.” | | | | NA | | None | A little | | | Moderate | | Great | | unable to do | |  |
| Q401 | Are you reading standard prints like a newspaper, books (Holy Bible, Quran)? | | | |  | |  |  | | |  | |  | |  | |  |
| Q402 | Reading large print (such as posters) and numbers on the telephone? | | | |  | |  |  | | |  | |  | |  | |  |
| Q403 | They recognize people when they are close to you? | | | |  | |  |  | | |  | |  | |  | |  |
| Q404 | Do you see curbs or steps? | | | |  | |  |  | | |  | |  | |  | |  |
| Q405 | Are you reading traffic signs, street sign, or store sign? | | | |  | |  |  | | |  | |  | |  | |  |
| Q406 | Are you doing fine handworks like sewing, knitting, or woodworking? | | | |  | |  |  | | |  | |  | |  | |  |
| Q407 | Are you doing farming activities like weed out and cutting? | | | |  | |  |  | | |  | |  | |  | |  |
| Q408 | Are you choosing and matching your clothes? | | | |  | |  |  | | |  | |  | |  | |  |
| Q409 | Are you participating in events and cultural sports(horse racing, hokey, spear)? | | | |  | |  |  | | |  | |  | |  | |  |
| Q410 | Cooking and other self-care activities (bathing)? | | | |  | |  |  | | |  | |  | |  | |  |
| Q411 | Are you watching television or photos? | | | |  | |  |  | | |  | |  | |  | |  |
| Q412 | Are you going out at day with bright sunlight? | | | |  | |  |  | | |  | |  | |  | |  |
| Q413 | Are you going out in a day with cloudy weather/at night? | | | |  | |  |  | | |  | |  | |  | |  |
| Q414 | Are you noticing objects off to the side while you are walking along? | | | |  | |  |  | | |  | |  | |  | |  |

| **Part 5: Willingness to pay for cataract surgery**  **Case Scenario**  **Introduction:** Cataract is the clouding of the eye’s natural lenses, and it is the leading cause of blindness worldwide. This problem can be caused by ageing, family history, hypertension, obesity, diabetes mellitus, smoking, significant alcohol consumption, and other courses. Cataract surgery is the only treatment option o solve this problem. Foreign donors fund this outreach program designed for cataract surgery. It aims to fight blindness due to cataracts by assuring the accessibility and equity of people living in districts. By now, you are getting these services free of charge because funding organizations cover the cost. However, in the future, this service will no longer be funded and sustainable  **Benefits of cataract surgery:**  Cataract surgery helps to improve vision, prevent avoidable blindness, increasing productivity, and improve quality of life.  As we have said before, to make the service sustainable and accessible, designing a cost-recovery model is very important. |
| --- |

After presenting the above case scenario, all participants were asked:

***Are you willing to pay some positive price for cataract surgery?***

1. ***Yes***
2. ***No***

If yes, follow the below algorism.





Iterative bidding technique elicits maximum willingness to pay for cataract surgery in outreach Sites, North West Ethiopia.

December 2018, Exchange rate: US$ 1=ETB 27.8.

**Thank you for being a part of this study!**
