# Supplementary material for: Willingness to pay for cataract surgery and associated factors among cataract patients in Outreach Site, North West Ethiopia
Source: PLoS One. 2021 Mar 24;16(3):e0248618. doi: 10.1371/journal.pone.0248618 (PMC7990211; doi:10.1371/journal.pone.0248618)
Supplement: Attachment — Submitted filename: PONE-D-19-31270_reviewer.pdf [file pone.0248618.s005.pdf]

PLOS ONE

Willingness to Pay for Cataract Surgery and Associated Factors among Cataract Patients in Outreach Site, North West Ethiopia

--Manuscript Draft--

Manuscript Number:	PONE-D-19-31270
Article Type:	Research Article
Full Title:	Willingness to Pay for Cataract Surgery and Associated Factors among Cataract Patients in Outreach Site, North West Ethiopia
Short Title:	Factors affecting willingness to pay for cataract surgery
Corresponding Author:	Getasew Amare<br>University of Gondar<br>Gondar, ETHIOPIA
Keywords:	
Abstract:	Introduction<br>In Ethiopia, cataract surgery is mainly being provided by donors free of charge through an outreach program. Planning a domestic source of finance is a fundamental scheme to sustain a program. Although knowledge concerning willingness to pay for cataract surgery is substantive for developing a cost-recovery model, the existed knowledge is limited and not well-addressed. Their fore, the study aimed to assess willingness to pay for cataract surgery and associated factors among cataract patients in Outreach Site, North West Ethiopia.<br>Methods<br>Institutional based cross-sectional study was conducted on 827 cataract patients, selected through simple random sampling method at Tebebe Gion Specialized Hospital from June 10 to 17/2018. Data were collected using a pretested structured questionnaire through face to face interview. The data was entered Epi info and exported to STATA 14 for analysis. The Tobit regression model was used to identify factors associated with willingness to pay. Regression coefficients (β), 95% confidence interval and p-value of <0.05 were used to measure the strength and presence of a statistical association.<br>Results<br>The study involved 827 cataract patients, with a response rate of 89%. Their median age was 65 years. About 55% of the participants were willing to pay for the cataract surgery and their average willingness to pay was 17.9 USD (95% CI; 10.79, 28.77). People in a state of still working, having formal education, being free from comorbidity, having unilateral cataract, and less time spent with the condition were significant factors for willing to pay.<br>Conclusion<br>Willingness to pay for cataract surgery in outreach Site is much lower than the actual cost of the surgery. Early intervention and developing cost-recovery model with multi-tiered packages attributed to the neediest people as in retired, less educated, severely disabled is strategic to increase the demand for service uptake and service accessibility.
Order of Authors:	Getasew Amare
	Mohammed Seid
	Amare Minyihun
	Gizachew Tilahun
	Asmamaw Ainafu
Additional Information:	
Question	Response
Financial Disclosure	This study was funded by University of Gondar. The views presented in the article are the authors and not necessarily express the views of the funding organization.

Powered by Editorial Manager® and ProduXion Manager® from Aries Systems Corporation

Enter a financial disclosure statement that describes the sources of funding for the work included in this submission. Review the submission guidelines for detailed requirements. View published research articles from *PLOS ONE* for specific examples.<br><br>This statement is required for submission and **will appear in the published article** if the submission is accepted. Please make sure it is accurate.<br><br>**Unfunded studies**<br>Enter: *The author(s) received no specific funding for this work.*<br><br>**Funded studies**<br>Enter a statement with the following details:<br>• Initials of the authors who received each award<br>• Grant numbers awarded to each author<br>• The full name of each funder<br>• URL of each funder website<br>• Did the sponsors or funders play any role in the study design, data collection and analysis, decision to publish, or preparation of the manuscript?<br>• **NO** - Include this sentence at the end of your statement: *The funders had no role in study design, data collection and analysis, decision to publish, or preparation of the manuscript.*<br>• **YES** - Specify the role(s) played.<br><br>* typeset	University of Gondar did not involve in the design of the study, data collection, analysis and interpretation.
**Competing Interests**<br><br>Use the instructions below to enter a competing interest statement for this submission. On behalf of all authors, disclose any competing interests that could be perceived to bias this work—acknowledging all financial support and any other relevant financial or non-financial competing interests.<br><br>This statement **will appear in the published article** if the submission is accepted. Please make sure it is accurate. View published research articles from *PLOS ONE* for specific examples.	Authors have declared that no competing interest exist.

**NO authors have competing interests**<br><br>Enter: *The authors have declared that no competing interests exist.*<br><br>**Authors with competing interests**<br><br>Enter competing interest details beginning with this statement:<br><br>*I have read the journal's policy and the authors of this manuscript have the following competing interests: [insert competing interests here]*<br><br>* typeset	
**Ethics Statement**<br><br>Enter an ethics statement for this submission. This statement is required if the study involved:<br><br>• Human participants<br>• Human specimens or tissue<br>• Vertebrate animals or cephalopods<br>• Vertebrate embryos or tissues<br>• Field research<br><br>Write "N/A" if the submission does not require an ethics statement.<br><br>General guidance is provided below. Consult the submission guidelines for detailed instructions. **Make sure that all information entered here is included in the Methods section of the manuscript.**	Ethical clearance was obtained from the ethical committee of Institute of Public Health, College of Medicine and Health Science, and University of Gondar. Official letter of permission was also obtained from Amhara Regional Health Bureau and Bahir Dar Tibebe Gion Specialized Referral Hospital. After explaining the purpose and importance of the study informed consent were obtained from each study participant. Participants were also informed that they have the right to withdraw from the study if they face any inconvenience during the interview processes

Powered by Editorial Manager® and ProduXion Manager® from Aries Systems Corporation

**Format for specific study types**<br><br>**Human Subject Research (involving human participants and/or tissue)**<br>• Give the name of the institutional review board or ethics committee that approved the study<br>• Include the approval number and/or a statement indicating approval of this research<br>• Indicate the form of consent obtained (written/oral) or the reason that consent was not obtained (e.g. the data were analyzed anonymously)<br><br>**Animal Research (involving vertebrate animals, embryos or tissues)**<br>• Provide the name of the Institutional Animal Care and Use Committee (IACUC) or other relevant ethics board that reviewed the study protocol, and indicate whether they approved this research or granted a formal waiver of ethical approval<br>• Include an approval number if one was obtained<br>• If the study involved *non-human primates*, add *additional details* about animal welfare and steps taken to ameliorate suffering<br>• If anesthesia, euthanasia, or any kind of animal sacrifice is part of the study, include briefly which substances and/or methods were applied<br><br>**Field Research**<br><br>Include the following details if this study involves the collection of plant, animal, or other materials from a natural setting:<br>• Field permit number<br>• Name of the institution or relevant body that granted permission	
**Data Availability**<br><br>Authors are required to make all data underlying the findings described fully available, without restriction, and from the time of publication. PLOS allows rare exceptions to address legal and ethical concerns. See the PLOS Data Policy and FAQ for detailed information.	Yes - all data are fully available without restriction

A Data Availability Statement describing where the data can be found is required at submission. Your answers to this question constitute the Data Availability Statement and **will be published in the article**, if accepted.<br><br>**Important:** Stating 'data available on request from the author' is not sufficient. If your data are only available upon request, select 'No' for the first question and explain your exceptional situation in the text box.<br><br>Do the authors confirm that all data underlying the findings described in their manuscript are fully available without restriction?	
**Describe where the data may be found in full sentences. If you are copying our sample text, replace any instances of XXX with the appropriate details.**<br><br>• If the data are **held or will be held in a public repository**, include URLs, accession numbers or DOIs. If this information will only be available after acceptance, indicate this by ticking the box below. For example: *All XXX files are available from the XXX database (accession number(s) XXX, XXX.*).<br>• If the data are all contained **within the manuscript and/or Supporting Information files**, enter the following: *All relevant data are within the manuscript and its Supporting Information files.*<br>• If neither of these applies but you are able to provide **details of access elsewhere**, with or without limitations, please do so. For example:<br><br>*Data cannot be shared publicly because of [XXX]. Data are available from the XXX Institutional Data Access / Ethics Committee (contact via XXX) for researchers who meet the criteria for access to confidential data.*<br><br>*The data underlying the results presented in the study are available from (include the name of the third party*	Data will be available from the corresponding author and it will be accessible to you when you want it.

Powered by Editorial Manager® and ProduXion Manager® from Aries Systems Corporation

and contact information or URL).<br>• This text is appropriate if the data are owned by a third party and authors do not have permission to share the data.<br><br>* typeset	
Additional data availability information:	

Powered by Editorial Manager® and ProduXion Manager® from Aries Systems Corporation

Manuscript Click here to download Manuscript manuscript.docx

Willingness to Pay for Cataract Surgery and Associated Factors among Cataract Patients in Outreach Site, North West Ethiopia

Mohammed Seid[1], Amare Minyihun[2], Getasew Amare[2*], Gizachew Tilahun[1], Asmamaw Ainafu[2]

Authors' Information

[1]Department of Optometry, School of Medicine, College of Medicine and Health Science, University of Gondar, Gondar, Ethiopia

[2]Department of Health Systems and Policy, Institute of Public Health, College of Medicine and Health Science, University of Gondar, Gondar, Ethiopia

MS: mohamiaseid@gmail.com

AM: amarebdr@gmail.com

GA: getasewa23@gmail.com

GT: gizachewtilahun1@gmail.com

AA: asmie2002@gmail.com

*Corresponding Author

E-mail: getasewa23@gmail.com(GA)

P.o Box: 196, University of Gondar, Gondar, Ethiopia

Abstract

Introduction

In Ethiopia, cataract surgery is mainly being provided by donors free of charge through an outreach program. Planning a domestic source of finance is a fundamental scheme to sustain a program. Although knowledge concerning willingness to pay for cataract surgery is substantive for developing a cost-recovery model, the existed knowledge is limited and not well-addressed. Their fore, the study aimed to assess willingness to pay for cataract surgery and associated factors among cataract patients in Outreach Site, North West Ethiopia.

Methods

Institutional based cross-sectional study was conducted on 827 cataract patients, selected through simple random sampling method at Tebebe Gion Specialized Hospital from June 10 to 17/2018. Data were collected using a pretested structured questionnaire through face to face interview. The data was entered Epi info and exported to STATA 14 for analysis. The Tobit regression model was used to identify factors associated with willingness to pay. Regression coefficients (β), 95% confidence interval and p-value of <0.05 were used to measure the strength and presence of a statistical association.

Results

The study involved 827 cataract patients, with a response rate of 89%. Their median age was 65 years. About 55% of the participants were willing to pay for the cataract surgery and their average willingness to pay was 17.9 USD (95% CI; 10.79, 28.77). People in a state of still working, having formal education, being free from comorbidity, having unilateral cataract, and less time spent with the condition were significant factors for willing to pay.

Conclusion

Willingness to pay for cataract surgery in outreach Site is much lower than the actual cost of the surgery. Early intervention and developing cost-recovery model with multi-tiered packages attributed to the neediest people as in retired, less educated, severely disabled is strategic to increase the demand for service uptake and service accessibility.

Introduction

Globally, cataract is the leading cause of blindness, which accounts for 51% of 39 million blind people (1, 2). In Ethiopia, 50% of blindness is also caused by a cataract (3). Cataract surgery is the only treatment modality (4, 5). According to Vision 2020 initiatives, the targeted CSR varies globally from 2000-5000(8). For African countries, it was projected to be 2000 per million per year but, now it is less than 500 (6). This underperformance was reasoned out by low patient demand, inadequate service delivery (6), insufficient family income (9, 10), costs of surgery (9, 11, 12), logistical constraints and fear of surgery (10, 12), lack of knowledge and lack of family support (12), lack of time (10). In 2005, the mean total costs per cataract intervention from nine European countries (Denmark, England, France, Germany, Hungary, Italy, Netherlands, Poland, and Spain) were US$ 843.5(13). By the year 2004 in the United States of America, the mean cost was estimated to be US$2525(14).

Moreover, evidence is entailing that actual cost exceeds the community's willingness to pay for cataract surgery (15, 16). Several studies showed that willingness to pay is affected by economic status, gender, age, family support, knowledge of the surgery, preoperative vision, family size, occupation, the level of perceived need, the decision-making processes at the family level and the characteristics of local eye care programs (15-23). A qualitative study also argues that eye care programs can influence WTP for cataract surgery by providing quality services and offering adequate counseling (23). Besides, many studies estimated that the mean amount of money as supportive evidence but the factors were not well identified (15, 16, 18, 20, 22, 24).

In Ethiopia, it is known that the eye care service delivery model is urban-centered, which is not easily accessible for rural dwellers. As a result, cataract surgery is being delivered for districts through outreach programs with the tremendous aid of donors in either free of charge or at a very low cost. The benefit of the program is preventing blindness through easily accessible and affordable strategy. As a result, the program can minimize the cost of blindness associated with cataracts. However, this might not be financially sustainable for the long-run. Therefore, knowledge concerning the actual cost of the surgery, the willingness to pay for the surgery and factors are substantive for developing a cost-recovery model that assures self-sustaining and high-volume surgical services.

Even if the knowledge of willingness to pay and the actual cost for cataract surgery is a necessity, the existed knowledge is not plenty to go through to the intervention. First, previous studies centered mainly on the patients' willingness to pay but not the amount. Moreover, the identified factors were for willingness pay as a proportion not to a specific amount. Second, willingness to pay is highly setting-variant. So it is less applicable beyond a study setting. Therefore, this study aimed to assess willingness to pay for cataract surgery and associated factors among cataract patients.

Methods

Study design and setting

Institutional Based cross-sectional study was conducted at Tebebe Gion Specialized Referral Hospital, Bahir Dar City, North West Ethiopia. This hospital is one of the tertiary hospitals in Ethiopia found in Bahir Dar city. This Hospital is expected to give service for around 5 million people.

Study population and sampling technique

All patients who admitted at Tebebe Gion specilaized Referral Hospital for cataract surgey were the study population. Participants who were under 18 and those who had communication barriers (speech impairment, hearing impairment) were excluded.

The sample size was determined using single population mean estimation formula, by assuming 95% confidence level, σ=standard deviation of WTP from previous study conducted Jimma, South west Ethiopia (US$7.5) (24), and It was considered to estimate the mean amount of money WTP within 0.5$ around the parameter at 95% confidence level. The final computed sample size including a 10% non-response rate was 926.

Screening for cataract was conducted in the nearby weredas before preparing the logistics of the surgery and the numbers of cataract patients prepared for the surgery were about 1336 patients. The average number of patients planned for the surgery per day was 170 cataract cases. During screening, cases were documented and appointed through giving identification

number. The Study participants were selected by using simple random method from daily appointed patients for cataract surgery.

Data collection procedures

Data were collected using a structured questionnaire through face to face interview. The questionnaire consists of socio-demographic/economic characteristics, health-related variables, preoperative visual function and hypothetical scenario about cataract surgery service. Double Bounded Dichotomous Choice Variant of the Contingent Valuation (CV) method was used estimate the participants' willingness to pay for the cataract surgery service interims of monetary value. The participants were asked the maximum amount they were willing to pay for the service. The questionnaire was pretested on 47 patients at Ebnat primary Hospital located which was the other outreach site.

Measurements and variables

Willingness to pay: Was the independent variable measured by questions prepared with bidding format. First, the participants were asked his/her willingness to pay something for the surgery. If the participant was willing to pay, he/she was asked WTP for the target price (ETB 1000 per eye), which was estimated from a study done in Southern Ethiopia in 2010 (28). The mean actual cost of cataract surgery per eye was 1911.2 ETB in 2010. So, the present value of the cost of cataract surgery per eye after 8 years was supposed to be 2824 ETB with an assumption of 5% discount rate. For a patient who accepted the first price, the bid was raised to 1250 ETB. If the participant again accepted this amount, the bid was raised to 1500 ETB. At this level, the participant was asked about the maximum WTP whether the participant accepted or rejected the 1500 bid. Correspondingly, if the participant rejected the starting bid

of 1000, the bid was lowered to 750 ETB. A participant who accepted this bid was asked about the maximum WTP. However, for a participant, who also rejected 750, the bid was decreased to 500 and then the participant was asked about the maximum WTP.

Wealth index: Household wealth was measured by asking a number of questions on the possession of different types of agricultural products, non-productive assets, and household facility. Wealth index quintile was derived by Principal Component Analysis (PCA).

Data processing and analysis

Then data were entered into Epi Info version 7 and was exported to Stata 14 for analysis. Data were coded and entered using Epi Info version7 software and exported to STATA version14 for analysis. Tobit econometric model was used to analyse the determinants of willingness to pay and the maximum amount of money that patients willing to pay

$$\text{y} = \begin{cases} 1 & i\ MWTP = \text{Bo} + \text{B'}\ Xi + \text{e} > 0 \\ 0 & if\ MWTP \leq 0 \end{cases}$$

Y = Outcome variable; MWTP = Maximum WTP; Xi = Explanatory variables; βo = Slope; β’ = Coefficient; ε = error term; 1 = Success/Yes; 0 = Failure/No

The model estimates marginal effect of an explanatory variable on the expected value of the dependent variable. The descriptive data were organized and presented using summary statistics, frequency distribution table and figures accordingly. To be free from serious data error the assumptions of Tobit model like normality and homoscedasticity of error terms through Breush-pagan for Heterosekedasticity test (X^2=1.78, p=0.18) and Multicollinearity via Variance inflation factor(VIF=1.23). Regression coefficients (β), 95% confidence interval and p-value of <0.05 were used to measure the strength and presence of statistical association.

Results

Participants' Sociodemographic and economic characterstics

A total of 827 participants involved in the study, which represented 89% response rate. The median age of the participants was 65 year with interqurtile range of 15. About 55% of the participants were age ≥65 years. Majority of the participants 498(60.2%) were males. Major portion of the participants 675(82%) were from rural and had no formal eduaction786(95%). Most of the participants were currenty married 515(62.2%) and about 84% of the particiapnts had family head role. About two-third of the participants 565(68.3%) were in state of still working (Table 1).

Table 1 Sociodemographic and economics characterstics of the study particiants for willingness to pay for cataract surgery and associated factors in outreach Site, North West Ethiopia, 2019 (n=827)

Variables	Frequency	Percent (%)
**Sex**		
Male	498	60.2
Female	329	39.8
**Age**		
<65 years	374	45.2
≥65years	453	54.8
**Residence**		
Rural	675	81.6
Urban	152	18.4
**Marital status(currently)**		
Married	515	62.3
Widow	229	27.7
Diveorced	63	7.6
Single	20	2.4
**Educational status**		
No formal education	786	95
Formal education	41	5

**Working state**		
Still working	565	68.3
Retired/unemployed	262	31.68
**Household head**		
Yes	695	84
No	132	16
**Family size**		
<5	586	70.86
≥5	241	29.14
**Wealth index quantiles**		
Poor	275	33.25
Meduim	276	33.77
Rich	276	33.77

Participants'health-related characteristics

Majority of the participants 638 (77.1%) were nor familarized withcataract surgery either from themselfeves or their family member. Nearly 714(86%) of the participants had unilateral operable cataract. Preoperatively, the particiapnts' mean visual ability score was 42.38 with standard deviation of 23. Majority of the participants 536(65%) had restricted visual performance, that was less than 50 visual ability score.The meantimespent with the condition was 2years. About 787(95%) of the participants did not report ocularcomorbidity other than cataract.Likewise, most of the participants 720(87%) did not reportanysystemic illness. More

than half of the participants 466(56.3%) stated that they were utilizing community based health insurance sevices(Table 2).

Table 2:Health-related characterstics of the study particiants for willingness to pay for cataract surgery and associated factors in outreach Site, North West Ethiopia, 2019(n=827)

Variables	Frequencey	Percent(%)
**Familiarity with cataract surgery**		
No	638	77.1
Yes	189	22.9
**Eye involved with cataract**		
One eye	714	86.3
Both eyes	113	13.7
**Preoperative Visual ability**		
<50	536	64.8
≥50	291	35.2
**Previous eye examination for cataract**		
No	634	76.7
Yes	193	23.3
**Time spent with condition**		
<2years	243	29.4
≥2years	584	70.6
**Self reported ocular morbidity**		
No	787	95.2
Yes	40	4.8

**Self reported Systemic illness**		
No	720	87.1
Yes	170	12.9
**Health insurance**		
Yes	466	56.3
No	361	43.7

Participants' willingness to pay for cataract surgery

Fifty five percent of the participants (456) were willing to pay something for the surgery and the average amount of money household heads willing to pay was 500ETB or 17.9USD (10.79 ,28.77). It's calculated as follows:-$AMWTP = \sum_{n=1}^{827}(\text{MWTP1} + \text{MWTP2} + \cdots .. + \text{MWTP927}) \div 827$

$= 500\ birr$ for cataract surgery service

Note: - 1$USD= 27.9 birr in May, 2019 currency exchange, MWTP is an average of willingness to pay; MWTP is the maximum amount of money willingness to pay.

Associated factors for willingness to pay for cataract surgery and their marginal effects

The study showed that the partcipants who were still working were willing to pay 26.66USD more than the partcipants who were retired or unemployed,keeping other variables constant,(β=26.66,95% CI 13.03, 40.29).The marginal effect of this variable revealed that changing working state from retired/unemployed to active working state increases WTP for cataract surgery by 14.46 USD from mean willingness to pay (dy/dx=14.46, 95% CI 7.48,

21.51). Participants who had formal education were WTP 29.16 USD more than partcipants who had no formal eduation (β=29, 16, 95%CI 2.35, 55.97).

Particiapnts who had no ocular comorbidity were WTP 28.48USD more than who had ocular comorbidity other than cataract, holding other variables constant, (β=28.48, 95%CI1.08, 55.90). The study depicted that being free from ocular morbidity increases WTP for cataract surgery by 14.26 USD from from mean willingness to pay (dy/dx=14.26, 95%CI 2.48, 26.04).

Likewise, participants who unilateral cataract were WTP 21.21USD more than those participants who had bilateral cataract, holding other variables constant (β=21.21, 95%CI3.65, 38.77). The marginal effect of this variable showed that only having unilateral cataract increases WTP for cataract surgery by 11.20 USD from mean willingness to pay (dy/dx=11.20, 95% CI 2.68, 19.73).

It was also depicted that one year increment in years spent with condition,the participants' WTP decreases by 1.69 USD, keeping the other variables constant,(β=-1.69,95%CI -3.32, -0.07).The marginal effect of this variable indicated that increasing years lived with the condition by one year, it may decrease the WTP for cataract surgery by 1.12 USD from the mean (dy/dx= -1.12, 95%CI -2.05, -0.19).

As the visual ability in the better eye increases by one unit, the particiapnts'WTP decrease by 0.29USD, (β=-0.29, 95%CI -0.55, -0.04) and the marginal effect of this variable revealed that increasing the visual ability in the better by one unit can decrease the WTP for cataract surgery by 0.16USD from mean willingness to pay (dy/dx=-0.16,95%CI-0.31,-0.02)(Table4)

Table 4:Maximum likelyhood of tobit econometric analysis of factors associated with WTP for cataract surgery in outreach Site,North West Ethiopia,2019(n=827)

Variables for MWTP	Category	Adjusted β coffcients	Standard error	t-value	p-value	95% CI Lower, upper	Dy/dx(95% CI)
Age	N	-0.34	0.26	-1.30	0.19	-0.85, 0.17	0.19(-0.48, .098)
Being Married (ref,single)	D	9.43	6.83	1.38	0.17	-3.98, 22.84	5.31(-2.14, 12.78)
Being Urban (ref,rural)	D	-0.77	8.24	-0.09	0.93	-16.95, 15.42	2.94 (-5.80, 11.68)
Formal Education(ref,noformal education)	D	29.16	13.66	2.14	**0.03***	2.35, 55.97	18.62(-0.22,37.46)
Still working (ref,retired/unemployed)	D	26.66	6.94	3.84	**0.000***	13.03, 40.29	**14.49 (7.48, 21.51)***
Being Family head(ref,No)	D	5.25	8.10	0.65	0.517	-10.65, 21.15	-0.44 (-9.61, 8.74)
Visual ability	N	-0.29	0.13	-2.26	**0.024***	-0.55, -0.04	**-0.16(0.31, -0.02)***
Time spent with the condition	N	-1.96	0.83	-2.37	**0.018***	-3.59, 0.34	**-1.12 (-2.05, -0.19)***
Familiarity with cataract surgery(ref,yes)	D	0.68	6.79	0.10	0.920	-12.67, 14.03	0.39 (-7.18, 7.96)
Previous eye examinations (ref,No)	D	6.50	6.71	0.97	0.33	-6.67, 19.68	3.77 (-3.97, 11.52)
No ocular comorbidity(ref,yes)	D	28.49	13.96	2.04	**0.04***	1.08, 55.90	**14.26 (2.48, 26.04)***
No systemic illness (ref,yes)	D	0.77	8.65	0.09	0.93	-16.2, 17.76	0.43 (-9.18, 10.06)
Involved eye with cataract(ref,both eyes)	D	21.21	8.95	2.37	**0.018***	3.65, 38.77	**11.20 (2.68,19.73)***

Meduim Wealth index(ref,poor)	D	-12.44	7.3	-1.70	0.089	-26.76, 1.89	-7.05(-15.16, 1.05)
Rich Wealth index(ref,poor)	D	-4.51	6.90	-0.65	0.51	-18.07, 9.04	-2.65 (-10.62, 5.31)
Having Health insurance(ref,No)	D	9.52	6.02	1.58	0.114	-2.28, 21.33	5.39 (-1.25, 12.04)
Family size	N	1.52	1.73	0.88	0.38	-1.87, 4.91	0.86 (-1.06, 2.79)
**Constant**		-29.02	28.07	-1.03	0.30	-84.12, 26.07	
**/Sigma**		73.08	2.68			67.82 78.34	

D, Dummy variable; N, numeric variable; ref, reference category;* significant with p-value <0.05

Discussion

This study aimed to assess WTP for cataract surgery and associated factors because this evidence is substantive for developing a cost-recovery model that assures self-sustaining and high-volume surgical services. Although more than half of the patients admitted for cataract surgery (55%) were willing to pay for the surgery, the average amount of money willing to pay for the surgery per eye was 500ETB (17.9USD).

This result was comparable with outreach based studies done in Jimma, Ethiopia (12.4USD (24) and in Northern Nigeria (18.5USD) (15). On the other hand, it is greater than the campaign based studies conducted in Tanzania (2.3USD (20), Malawi (3USD), (16) and Nepal (7USD) (21). Even if the studies were outreach based, there were conducted on a very small sample size selected with non-probability sampling. This may be the reason for the discrepancy. However, this result was lower than the studies done in Hong Kong (552USD) (17) and China (968USD) (22). Both studies involved cataract patients on a waiting list in Hospitals and biding format was used for eliciting the willingness to pay after setting the target prices based on estimated market price. Relatively, their target price was higher as compared to the present study. Having differences in target price and research setting might be taken as a reason for the difference.

Overall, this amount of money willing to pay matches the subsidized price for cataract surgery at General and Tertiary Hospitals but it was significantly less than the current cost of the surgery in the area, 2824 ETB(101.2USD) (28). Lack of money, lack of knowledge to access the services and waiting for the campaign were the main reason cited to be unwilling to pay and unable to seek eye care services previously.

Currently, cataract surgery is being delivered for districts through outreach programs with the tremendous aid of donors in free of charge. However, this might not be financially sustainable for the long-run. The findings of this study imply that if the government or private sectors plan to sustain the program with domestic resources and increase the cataract surgical coverage and uptake, it is strategic to develop an inclusive cost-recovery model and health promotion that can assure services accessibility, self-reliance, and equity. The Aravind Eye Care System in India is the best lesson, which is providing high-quality, and high-volume (350,000 eye operations per year) services to those who can afford to pay market rates and then uses the profits to fund care for those who cannot. Patients who cannot afford to pay are given cataract surgery for free. However, the government reimburses Aravind 10USD for each procedure (25). The Ethiopian health care financing policy considers the systematizing fee waiver system, while such approaches are not applied for cataract surgical services so far.

Moreover, there is a probability of integrating the service in the existed health care system at the district level and sustaining with domestic resources if the Ethiopian government invests on human resource development and infrastructure because this study revealed that WTP is comparable with the current price of cataract surgery done at General and Tertiary Hospitals.

It is also of interest to note about the impact of working state on willingness to pay that the participants who were still working were WTP 26.66USD more than the participants who were retired/unemployed. This result is supported by a study conducted in rural china (19) though some studies concluded that willingness to pay was not affected by the working state (16, 22, 26). This relationship can be explained in a way that being employed can be related to greater productivity. This implies that retired/ unemployed persons would be potential targets for cost-recovery.

Persons with formal education were 29.16USD more WTP than those who had no formal education for surgery. This finding is consistent with a study done in China (18). This may suggest that educated persons may value their vision more and may have good knowledge about the benefit of the procedure and even the cost of the service. However, many studies found that WTP for cataract surgery was not significantly affected by literacy because these studies were done in nations where there is higher education coverage (17, 19, 24, 26). Hence, significant variation based on education might not be observed. This result indicated that extra efforts in outreach programs need to be invested to prosper the knowledge of illiterate persons in non-printed media and provision of cataract surgical services by offering multi-tiered package attributed to literacy.

Patients who had no ocular comorbidity and unilateral cataracts were more WTP than who had ocular comorbidity and bilateral cataract. Previous studies reported similar findings, which preexisting eye diseases other than cataract and bilateral cataract affects willingness to pay for cataract surgery (19,22). This may be explained in a way that persons with comorbidity and bilateral cataract might have poor visual performance. Hence, their productivity and economy may be compromised, which may desperate them to have good vision and uptake the services. On the other perspective, as the visual performance of better eye decreases by one unit, the participants' WTP increases by 0.29USD. Similarly, a study reported that persons with visual function scores below 80(poor visual performance), were more willing to pay for the surgery (17). The justification might be that patients with bilateral cataracts can be highly ambitioned to enhance their daily base activities than those who have good vision in the better eye regardless of their ability to pay. However, two studies found that as the preoperative VA becomes more impaired, WTP decreases, in which the visual functionality was measured

objectively (16, 18). This finding implies that subsidization of the cost of the surgery is substantive for patients with severe visual disability-related to the preexisted condition and bilateral lens opacification. Thus, even if the efficiency of resource utilization is sacrificed, equity among services consumers might be achieved.

The study also depicted that one-year increment in years lived with visual impairment, the participants WTP decreases by 1.69USD. The explanation might be that as the duration increase without intervention, the severity of visual disability and complication associated with cataract can be increased. This increases the psycho-socio-economic burden of the condition and it ends up with a poor vision-related quality of life. Hence, the ability to pay for a service might be questionable and WTP as well. This implies that early intervention may promote a greater willingness to pay and utilization of the services.

However, Participants' residence, sex, family size, age, family head role, marital status, familiarity with cataract surgery, systemic illness and health insurance, were not significantly associated with WTP for cataract surgery. Similar findings were reported by many previous studies (16-20, 22, 26, 27).

Although the application of abiding eliciting approach with well-described case scenario and drawing cases from different districts are considered as strengths, it must be understood within the context of some hypothetically induced overestimation of willingness to pay. This study also did not consider enhancement packages for willingness to pay and willingness to pay was not tailored with the consumers' ability to pay for the services. It is also conceived that the findings would be augmented with qualitative research to capture reasons for unwilling to pay for cataract surgery in detail.

Conclusion

Willingness to pay for cataract surgery among adults with operable cataract in outreach Site is much lower than actual cost of the surgery. People with state of having formal education, working state; less time spent with the condition, free from co morbidity and bilateral cataract were more willing to pay for cataract surgery.

List of abbreviations

CV = Contingent Valuation, EDHS = Ethiopian Demographic and Health Survey, ETB = Ethiopian Birr, GDP = Gross Domestic Product, HEP = Health Extension Program, HEW = Health Extension Worker, HP = Health Post, HSDP = Health Sector Development Program, MWTP = Maximum Willingness to Pay

Declaration

Ethical approval and consent to participate

Ethical clearance was obtained from the ethical committee of Institute of Public Health, College of Medicine and Health Science, and University of Gondar. Official letter of permission was also obtained from Amhara Regional Health Bureau and Bahir Dar Tibebe Gion Specialized Referral Hospital. After explaining the purpose and importance of the study informed consent were obtained from each study participant. Participants were also informed that they have the right to withdraw from the study if they face any inconvenience during the interview processes.

Funding

This study was funded by University of Gondar. The views presented in the article are the authors and not necessarily express the views of the funding organization. University of Gondar did not involve in the design of the study, data collection, analysis and interpretation.

Consent to publish

Not applicable

Authors' contribution

MH conceived the study, developed the tool, coordinated the data collection activity, and carried out the statistical analysis. AM participated in the design of the study, development of the tool, and drafting of the manuscript. GA participated in the design of the study and development of the tool, performed statistical analysis and drafted the manuscript. GT participated in development of the tool and manuscript development. All authors read and approved the final manuscript.

Availability of data and materials'

Data will be available upon request from the corresponding author.

Acknowledgements

Authors would like to thank all respondents for their willingness to participate in the study. We are also grateful to Amhara public health institute, Tebebe Gion Specialized Referral Hospital, and University of Gondar for material support. Finally, our appreciation goes to data collectors for their unreserved contribution in data collection activities.

Competing interests

Authors declare that they have no conflict of interest.

References

1.World Health Organization. Global data on visual impairments; 2010. Available from:http://wwwwhoint/blindness/publications/globaldata/en/.Accessed on 7/13/2018.

2. Foster A, Gilbert C, Johnson G. Changing patterns in global blindness: 1988–2008. Community Eye Health. 2008;21(67):37.

3. Berhane Y, Worku A, Bejiga A, Adamu L, Alemayehu W, Bedri A, et al. Prevalence and causes of blindness and low vision in Ethiopia. Ethiopian Journal of Health Development. 2007;21(3):204-10.

4. Lumme P, Laatikainen LT. Factors affecting the visual outcome after cataract surgery. International ophthalmology. 1993;17(6):313-9.

5. Huang W, Huang G, Wang D, Yin Q, Foster PJ, He M. Outcomes of cataract surgery in urban southern China: the Liwan Eye Study. Investigative ophthalmology & visual science. 2011;52(1):16-20.

6. Fostera A. Cataract and “Vision 2020:the right to sight” initiative. British Journal of Ophthalmology. 2001;85(6):635-7.

7. Fang J, Wang X, Lin Z, Yan J, Yang Y, Li J. Variation of cataract surgery costs in four different graded providers of China. BMC Public Health. 2010;10:543.

8. Lewallen S, Roberts H, Hall A, Onyange R, Temba M, Banzi J, et al. Increasing cataract surgery to meet Vision 2020 targets; experience from two rural programmes in east Africa. British Journal of Ophthalmology. 2005;89(10):1237-40.

9. Mehari ZA, Zewedu RTH, Gulilat FB. Barriers to cataract surgical uptake in central ethiopia. Middle East African journal of ophthalmology. 2013;20(3):229.

10. Snellingen T, Shrestha B, Gharti M, Shrestha J, Upadhyay M, Pokhrel R. Socioeconomic barriers to cataract surgery in Nepal: the South Asian cataract management study. British Journal of Ophthalmology. 1998;82(12):1424-8.

11. Zhang M, Wu X, Li L, Huang Y, Wang G, Lam J, et al. Understanding barriers to cataract surgery among older persons in rural China through focus groups. Ophthalmic epidemiology. 2011;18(4):179-86.

12. Jadoon Z, Shah SP, Bourne RR, Dineen B, Khan MA, Gilbert CE, et al. Cataract prevalence, cataract surgical coverage and barriers to uptake of cataract surgical services in Pakistan: the Pakistan National Blindness and Visual Impairment Survey. British Journal of Ophthalmology. 2007;91:1269–1273.

13. Fattore G, Torbica A. Cost and reimbursement of cataract surgery in Europe: a cross-country comparison. Health Economics. 2008;17(1):71-82.

14. Lansingh VC, Carter MJ, Martens M. Global cost-effectiveness of cataract surgery. Ophthalmology. 2007;114(9):1670-8.

15. Ibrahim N, Ramke J, Pozo-Martin F, Gilbert CE. Willingness to pay for cataract surgery is much lower than actual costs in Zamfara state, northern Nigeria. Ophthalmic epidemiology. 2018;25(3):227-33.

16. Dean WH, Sherwin JC, Kumwenda S, Angeletti M, Wiehler U. Willingness to pay for cataract surgery in post-operative cataract patients in rural Malawi. Ophthalmic epidemiology. 2012;19(5):265-71.

17. Chan FW-k, Fan AH, Wong FY-y, Lam PT-h, Yeoh E-k, Yam CH-k, et al. Waiting time for cataract surgery and its influence on patient attitudes. Investigative ophthalmology & visual science. 2009;50(8):3636-42.

18. He M, Chan V, Baruwa E, Gilbert D, Frick KD, Congdon N. Willingness to pay for cataract surgery in rural Southern China. Ophthalmology. 2007;114(3):411-6.

19. Ko F, Frick KD, Tzu J, He M, Congdon N. Willingness to pay for potential enhancements to a low- cost cataract surgical package in rural southern China. Acta ophthalmologica. 2012;90(1):54-60.

20. Lewallen S, Geneau R, Mahande M, Msangi J, Nyaupumbwe S, Kitumba R. Willingness to pay for cataract surgery in two regions of Tanzania. British journal of ophthalmology. 2006;90(1):11-3.

21. Shrestha M, Thakur J, Gurung C, Joshi A, Pokhrel S, Ruit S. Willingness to pay for cataract surgery in Kathmandu valley. British journal of ophthalmology. 2004;88(3):319-20.

22. Wang M, Zuo Y, Lin X, Ling Y, Lin X, Li M, et al. Willingness to pay for cataract surgery provided by a senior surgeon in urban Southern China. PloS one. 2015;10(11):e0142858.

23. Geneau R, Massae P, Courtright P, Lewallen S. Using qualitative methods to understand the determinants of patients' willingness to pay for cataract surgery: a study in Tanzania. Social science & medicine. 2008;66(3):558-68.

24. Gessesse G, Demissie B. Willingness to Pay for Cat-aract Surgery Among Outreach Site Patients in South West Ethiopia: A Cross-Sectional Study. Int J Ophthalmol Eye Res. 2014;2(6):65-9.

25. Aravind Eye System. Driving down the cost of high-quality care:Lessons from the Aravind Eye Care System. at https://www.mckinsey.com. Accessed on 7/13/2018.

26. Radhakrishnan M, Venkatesh R, Valaguru V, Frick KD. Household preferences for cataract surgery in rural India: a population-based stated preference survey. Ophthalmic epidemiology. 2015;22(1):34-42.

27. Kessy JP, Lewallen S. Poverty as a barrier to accessing cataract surgery: a study from Tanzania. British journal of ophthalmology. 2007;91(9):1114-6.

28. Habtamu E, Eshete Z, Burton MJ. Cataract surgery in Southern Ethiopia: distribution, rates and determinants of service provision. BMC health services research. 2013;13:480.
