# Supplementary material for: Willingness to pay for cataract surgery and associated factors among cataract patients in Outreach Site, North West Ethiopia
Source: PLoS One. 2021 Mar 24;16(3):e0248618. doi: 10.1371/journal.pone.0248618 (PMC7990211; doi:10.1371/journal.pone.0248618)
Supplement: Attachment — Submitted filename: Response to reviewers.pdf [file pone.0248618.s006.pdf]

Response reviewer

Reviewer 01

Question 1.

The manuscript (including the abstract) demands massive improvement in writing. Moreover, there are typos and grammatical errors that need to be corrected.

Authors' response

Dear reviewer thanks for your comments and questions.

We tried to look and revise the whole document based on the given comments.

Question 02

WTP are health-state dependent, hence, it depends on the context and techniques applied (e.g. double bounded dichotomous choices) to elicit WTP. Sharing the tool (as supplementary documents) might be a good to option to let the reader understand the WTP estimation techniques applied in this study.

Authors' response

Dear reviewer thanks for your comments and questions.

We tried to include the WTP elicit schematic diagram on page 9.

Question 03

Generally, outreach-sites are socio-demographically different from the general population. A comparison of socio-demographic characteristics between the sample and general population of North-West-Ethiopia or Ethiopia might provide a better understanding of the sample as-well-as understanding the differences in WTP in a general context.

Authors' response

Dear reviewer thanks for your comments and questions.

It is rewritten in a way that describes socio-demographic characteristics of the study population and for whom the study would be inferred. Stated in Page 6 as "***Campaign Based cross-sectional study was conducted and the site was at Tebebe Gion Specialized Referral Hospital, Bahir Dar city, North West Ethiopia. There are only four Tertiary and general hospitals for about 14 million people in the catchment area, which provide comprehensive eye care services including cataract surgical services in North West Ethiopia. However, all of them are urban-centered and not accessible for more than 80% of the community. Hence, the outreach program was planned for communities where cataract surgical services are not available and for peoples in the disadvantaged area with low socio-economic status in North West Ethiopia. Initially, screening for cataracts was conducted in the nearby rural weredas before the campaign. During the screening, Cataract cases were diagnosed and informed about the condition and scheduled to attend the planned outreach program. Hence, the cases were selected and linked to the campaign from weredas of West Gojjam, South Gondar, and Awi Zone. A total of 1336 cases (628 cases from West Gojjam, 374 cases from South Gondar, 201cases from Awuyi zone, 100 cases from Bahir Dar City, and 33 cases from East Gojjam) were admitted to the outreach Site. Thus more than 93% of cases were from rural weredas and district towns. Therefore, the findings of this study could apply to rural residents admitted for cataract surgery in outreach programs***"

Question 04

The 'Introduction' mentions the mean costs of cataract surgery in Europe and USA. The cost of the surgery varies across the globe. As the study estimates the WTP for cataract surgery in Ethiopia, it would be wise to also state literatures that estimate WTP of other African and Asian countries (similar economies).

Authors' response

Dear reviewer thanks for the comments and questions.

It is rewritten in pages 4-5 as "***Moreover, studies from Africa and South East Asia reported that the actual cost exceeds the community's willingness to pay for cataract surgery (15-20). Overall, the proportion of WTP in Asia was very discrepant, which was ranged from 34% to 90%. Similarly, the mean amount of money willing to pay was significantly varied from US$ 7 to US$ 968 (17, 19, 21, 22, 26). However, the mean amount of money WTP in Africa was ranged from US$2.3 to US$18.5, which was low as compared to Asian nations (15, 16, 20, 24)***

Question 05

The 'Introduction' ends with a non-justifiable argument (Please check the sticky-note-comment). The manuscript states about the context-dependent WTP, however, it does not state any literature on context or health-state dependencies of estimated WTP. Also, please provide a detailed description of the context (i.e. WTP elicitation approach) in the method section.

Authors' response

Dear reviewer thanks for the comments and questions

It is stated on page 5 as "***Even if the knowledge regarding willingness to pay and the actual cost of the surgery is essential, the existed knowledge is not plenty to go through the intervention. Firstly, the existed knowledge in the study area is mainly centered on the proportion of willingness to pay rather than the amount of money (23). Secondly, the factors for the amount of money WTP were not fully addressed with the appropriate analysis model. Thirdly, willingness to pay is highly setting-variant, which mainly varies with socioeconomic status, and study setting. Consequently, it is difficult to apply the research finding done in other corners of the globe for this study area. Therefore, this study aimed to assess the willingness to pay for***

cataract surgery and associated factors among cataract patients at the outreach site in Northwest Ethiopia’’.

Question 06

There are various surgical techniques of cataract surgery (e.g. Small incision cataract surgery, phacoemulsification). For which technique of surgery respondents elicited their WTP for? Did they have full information or any prior knowledge of the surgical procedure(s)?

Authors’ response

Dear reviewer thanks for your comments and questions.

It is stated on page 8 as “***First, the participants were asked his/her willingness to pay something for the Manual Small incision cataract surgery***.” Regarding prior information, they have been informed during screening, which is stated on page 6, as ‘***During the screening, Cataract cases were diagnosed and informed about the condition…..***.”

Question 07

The ‘Study design and setting’ section should focus on the eye care provided by the hospital. Please provide a summary of the eye care facilities available in North West Ethiopia. It will help the reader to understand the representativeness of the setting (i.e. hospital) from which respondents were selected.

Authors’ response

Dear reviewer thanks for your comments and questions.

It is written on page 6 as ***an “Outreach- based cross-sectional study was conducted. The campaign site was at Tebebe Gion Specialized Referral Hospital, Bahir Dar city, North West Ethiopia. There are only four Tertiary and general hospitals, for about 14 million people in the catchment area, which provide comprehensive eye care services including cataract surgical***

services in North West Ethiopia. However, all of them are urban-centered and not accessible for more than 80% of the community. Hence, the outreach program was planned for communities where cataract surgical services are not available and for peoples in disadvantaged areas with low socioeconomic status in North West Ethiopia. Initially, screening for cataracts was conducted in the nearby rural weredas, which are the third-level administrative units in the Ethiopian governance structure, before the campaign. During the screening, Cataract cases were diagnosed and informed about the condition and scheduled to attend the planned outreach program. As a result, a total of 1336 cataract cases were linked and admitted to the campaign from weredas of West Gojjam(628cases), South Gondar (374cases) Awi Zone (201 cases), Bahir Dar City (100 cases) and from East Gojjam (33cases). Thus more than 93% of cases were from rural weredas and district towns. Therefore, the findings of this study could apply to rural residents admitted for cataract surgery in outreach programs."

Question 08

The 'Study population and sampling technique' section need to improve its clarity in describing the sampling technique used for the study. A simple random sampling technique requires selecting subjects from a predetermined sampling frame. If the list of 1336 cataract patients was used as the sampling frame, then at least two things need to be clarified.

1. The sampling frame was prepared based on the patients who visited the facility from June 10, 2018, to June 17, 2018; were the subjects chosen daily or based on an aggregated list.

Authors' response

Dear reviewer thanks for the comments and questions.

It is rewritten on page 6 as ***"Initially, screening for cataracts was conducted in the nearby rural weredas, which are the third-level administrative units in the Ethiopian governance structure,***

before the campaign. During the screening, Cataract cases were diagnosed and informed about the condition and scheduled to attend the planned outreach program. As a result, a total of 1336 cataract cases were linked and admitted to the campaign from weredas of West Gojjam(628cases), South Gondar (374cases) Awi Zone (201 cases), Bahirdar City (100 cases) and from East Gojjam (33cases).

Thus more than 93% of cases were from rural weredas and district towns. Therefore, the findings of this study could apply to rural residents admitted for cataract surgery in outreach programs.''

1. As the program provides free of cost cataract surgery, the subjects interviewed in this study should already have a preconceived estimation about the cost of the treatment; this has a definite impact on the elicited WTP (e.g. only 55 percent respondents elicited a non-zero WTP).

Authors' response

Dear reviewer thanks for the comments and questions.

We have developed a case Scenario to minimize information asymmetry regarding cataract surgery and to estimate how beneficiaries value this service rationally. Even if we use the **Case scenario to minimize strategic bias, the WTP approach is deemed not free of the afro-mentioned problem. So we have stated it in the limitation section.** we have attached the case scenario at the supplementary file with the data collection tool.

Case Scenario

" ***Introduction: Cataract is clouding of the eyes natural lenses and it is the leading cause of blindness worldwide. This problem can be caused by aging, family history, hypertension, obesity, diabetes mellitus, smoking, significant alcohol consumption, and other courses. To overcome***

this problem cataract surgery is the only treatment option. This outreach program which is designed for cataract surgery is funded by foreign donors and aims to fight blindness due to cataract by assuring accessibility and equity of the services for people living in districts. You are getting these services by now free of charge, but this is because of the cost is covered by funding organizations. However, in the future, this service will no longer be funded and sustainable.

Benefits of cataract surgery:

The cataract surgery helps to improve vision, prevent avoidable blindness, increasing productivity, and improve quality of life. As we have said before to make the service sustainable and accessible, designing a cost-recovery model is very important.

Question 09

The description of the bidding procedure given on page 5 needs clarity. How was the initial bid (i.e. 1000 ETB) was chosen? A schematic representation of the DBDC experiment would help the reader to visualize the context better

Authors' response

Dear reviewer thanks for your comments and questions.

The biding experiment is put on ***page 9*** now. It is clarified again as follows on page 8, as **"*The starting price for the biding game was estimated from the actual cost of manual small incision cataract surgery per eye, which was 2824 ETB from a study done in Southern Ethiopia in 2010 (34). In the Ethiopian context, the average household out pocket expenditure from the total health financing is about 35%. Based on this rate, the starting price that we expected to be paid was 1000 Birr (0.35x2824birr). During eliciting, the participants were asked their willingness to pay something for the Manual Small incision cataract surgery initially. If the participant was willing to pay, he/she was asked WTP for the target price (ETB 1000 per eye).''***

Question 10

About 45 percent of respondents were not willing to pay a positive price for the service, how were these responses handled in the analysis? Besides, if some people were not willing to pay, were these people statistically different from those that were willing to pay?

Authors' response

Dear reviewer thanks for the comments and questions.

To handle those participants who are not willing to accept to pay any price for the cataract surgery, Tobit regression econometric model was used. This model also considered as censored regression model used to estimate the parameters of how explanatory variables affect the latent dependent variable. So, 45 % of participants who are considered as censored for the lower limit were included in the model with an analysis of the Tobit regression model.

Question 11

The DBDC experiment ends with an interval of WTP. Did you check the interval regression techniques to estimate the associated factors? (If yes) Were the results different from an interval regression model?

Authors' response

Dear reviewer thanks for the comments and questions.

We are using the Contingent valuation method with a bidding game elicitation approach to explore the participants' maximum willingness to pay for cataract surgery. The participants were asked as biding up or down based on their responses to the preceding price. The end question given for the participants was not Yes/No, rather an open question about their maximum willingness to pay. So, the final response for all participants who are willing to pay any positive price for cataract surgery was open continues price, not kind of Yes/No answer for

the stated price. We only use those sated prices to elicit and manage the biding game to get the maximum value participants will give for the service. Due to this, we didn't run the interval regression to identify associated factors.

Question 12

The average WTP can be estimated using a constant only interval regression model. Although, 45 percent respondents did not elicit a positive WTP, their true WTP might be greater than zero but less than the last bid (750 ETB). In that case counting them as zero (as done in this manuscript) might not be pragmatic

Authors' response

Dear reviewer thanks for the comments and questions.

After presenting the scenario the initial question raised for the participants was as they willing to accept some price for the cataract surgery or not. So, those who were not willing any amount of payment for the cataract surgery (around 45% of the participants) waived the next biding game which was used to explore the maximum amount of price they want to pay for the service. The biding game approach was only applied to those participants (55%) who were willing or volunteer to pay some price for the service. Those participants who were not willing to pay any positive price were considered as a censored sample.

Question 13

The robustness of the Tobit models can be checked estimating coefficients of both censored and truncated samples as-well-as estimating the OLS coefficients. Please report the results (as supplementary documents). This practice will help the readers understand the robustness of the results obtained by the study.

Authors' response

Dear reviewer thanks for the comments and questions.

We have estimated the coefficients using the censored (Tobit) regression model, truncated regression model, and using the OLS model and attached as a supplementary file.

Question 14

The 'Participant's health-related characteristics' section requires to provide sufficient details of the variables (what and how the measurements were taken; e.g. Systemic illness).

Authors' response

Dear reviewer thanks for the comments and questions.

The respondents were asked about their self-reported systemic illness.

Question 15

The *discussion* and *conclusion* need to be improved.

Authors' response

Dear reviewer thanks for your comments and questions.

We tried to revise and rewrite the discussion and the conclusion.

Reviewer 02

Question 01

The overall standard of academic English is low and one has to struggle to read between the lines to understand the otherwise important information being conveyed. May I suggest the authors seek professional assistance is preparing the grammatical content of the manuscript.

Authors' response

Dear reviewer thanks for your comments and questions.

We have revised the manuscript both grammatically and content-wise.

Question 02

Many of the references are out of date. There are more recent references on the prevalence and causes of blindness, globally.

Authors' response

Dear reviewer thanks for your comments and questions

We have revised the references in the main document

Question 03

In the Methods section, the authors state that all patients admitted for cataract surgery at Tebbe Gion Hospital formed the study population, then mentions that the patients were screened for cataract. What was the purpose of the screening? When was this study conducted?

Authors' response

Dear reviewer thanks for your comments and questions.

Dear reviewer thanks for the comments. We have put detailed information on how and why cataract pre-screening was done and when was the study conducted on pages 6 and 7. We put it as follows.

"An Outreach- based cross-sectional study was conducted. The campaign site was at Tebebe Gion Specialized Referral Hospital, Bahirdar city, North West Ethiopia. There are only four Tertiary and general hospitals, for about 14 million people in the catchment area, which provide comprehensive eye care services including cataract surgical services in North West Ethiopia. However, all of them are urban-centered and not accessible for more than 80% of the community. Hence, the outreach program was planned for communities where cataract surgical services are not available and for peoples in disadvantaged areas with low socioeconomic status in North West Ethiopia. Initially, screening for cataracts was conducted in the nearby rural weredas, which are the third-level administrative units in the Ethiopian governance structure,

before the campaign. During the screening, Cataract cases were diagnosed and informed about the condition and scheduled to attend the planned outreach program. As a result, a total of 1336 cataract cases were linked and admitted to the campaign from weredas of West Gojjam(628cases), South Gondar (374cases) Awi Zone (201 cases) Bahirdar (100 cases) and from East Gojjam (33cases). Thus more than 93% of cases were from rural weredas and district towns. Therefore, the findings of this study could apply to rural residents admitted for cataract surgery in outreach programs.''

Question 04

Several unfamiliar analytical terms are mentioned.

Double Bounded Dichotomous Choice Variant of the Contingent Valuation (CV)

Principal Component Analysis (PCA)

Homoscedasticity of error terms through Breush-pagan for Heterosekedasticity test

The authors should briefly explain what they are and put a reference as this is a broad remit medical journal

Authors' response

Dear reviewer thanks for your comments and questions.

We have explained these terms in the main document method part and we have also included the reference.

Question 05

We didn't measure visual acuity rather Visual ability which is the overall visual performance including visual acuity, color vison, contrast sensitivity, and visual field. So, the visual ability was measured using modified VF-14 tool, not Snellen's notation or the log MAR.

Reviewer 03

Question 01

I found it quite difficult to understand the text because English is not written in standard syntax and grammar. I strongly suggest that the authors engage the service of a professional English editing service before re-review of the manuscript

Authors' response

Dear reviewer thanks for your comments and questions

We have revised the manuscript both content-wise and grammatically.

Question 02

Besides, the methods and statistics require some more explanation for clarity and ease of understanding by the reader.

Authors' response

Dear reviewer thanks for your comments and questions.

We explain those terms like WTP, CV method, Bidding game elicitation approach, and Tobit regression econometric model at the method section.
