# Supplementary material for: Willingness to pay for cataract surgery and associated factors among cataract patients in Outreach Site, North West Ethiopia
Source: PLoS One. 2021 Mar 24;16(3):e0248618. doi: 10.1371/journal.pone.0248618 (PMC7990211; doi:10.1371/journal.pone.0248618)
Supplement: Attachment — Submitted filename: Response to Reviewers.pdf [file pone.0248618.s010.pdf]

Response to Reviewers

Reviewer 1 comments and questions: -

1. Please mention the dates of data collection also in the main body.

Author response

Dear reviewer, thanks for your comments and questions

We have revised the manuscript as per the given comment. Please see page 7 and line 136 of the revised manuscript.

2. The exchange rate used for the survey is the one of 2018. However, the world bank data the manuscript refers to in line no. 149 on page 8 is of the year 2009 (see reference no. 43). Moreover, the manuscript mentions that the out of pocket health expenditure is 35 percent of the total health expenditure in Ethiopia. Whereas, the report it refers to in reference no. 43 shows that it is 37 percent for Ethiopia. Authors should be very transparent and careful about the methodologies used and the methodologies described in the manuscript.

Author response

Dear reviewer, thanks for your comments and questions

We have revised the reference, and we have removed the previous reference, and we have added the world bank group 2017 report of Ethiopia and Sub-Saharan African countries. Please see page 8 and lines 148 to 150 of the revised manuscript.

3. Line no. 152-154 mentions that the respondents were asked – "*Are you willing to pay some positive price for cataract surgery?*", however, the questionnaire attached as a supplementary file states that, "*by considering the above scenario, if the service is vailing for you, are you willing to pay something for this service*". Although, the sentences provide the same

understanding of the question, there should not be any inconsistencies between what is written in the manuscript and what was actually asked. If the author wants to describe this as part of the methodology they applied; then, the exact question need not to be mentioned. This could be written differently, such as, "*After briefing the case scenario, all the participants were asked whether they are willing to pay any positive price for the proposed cataract surgery*".

Author response

Dear reviewer, thanks for your comments and questions

We have reviewed it, and we have made it consistent have was written on the methodology and the tool part.

4. The intervals are not correctly specified in the schematic diagram. If anyone is not willing to pay 500 ETB, then the interval they fall into is 0 to <500 ETB. The intervals should be fixed by the experiment-design, not the average maximum WTP.

Author response

Dear reviewer, thanks for your comments and questions.

We tried to revise the intervals in each experimental design. Please see page 9 figure of the revised manuscript.

5. The dataset still does not contain the bidding experiment data. Sharing partial dataset will not help serving the right purpose of data-sharing.

Author response

Dear reviewer, thanks for your comments and questions

We have included the biding experiment data in the revised data set. Please see the revised data set.

Reviewer 2 comments and questions: -

1. The authors have addressed some of my comments. The main concern I had and still have is about the visual ability measurement. This is not a regular term and the authors have not defined it or explained how it was assessed. The crux of the paper is the relationship between visual ability and WTP, so this is a major flaw which the authors have not addressed. Visual acuity is usually used for WTP research. The references provided about visual ability refer to visual acuity.

Authors response

Dear reviewer, thanks for your comments and questions

We have accepted your comments, and we measured pre-operative visual ability by using the Visual Function 14 Index (VF-14) tool. The response option was prepared with Likert's scale format. The scale ranged from the 0-4 value for the degree of visual difficulties 'unable to do' (scale 0), 'great difficulty' (scale1), 'moderate difficulty' (scale 2), 'little difficulty' (scale 3) and 'no difficulty' (scale 4). Then each scale is multiplied by 25. So, the value of each response ranges from 0-100. After that, the factored amounts were summed up. Finally, VF index was computed by dividing the summed factored amounts to the number of checked boxes. Visual ability was categorized as normal or near-normal performance (≥50 visual ability score) and restricted performance (<50 visual ability score) based on the international council of Ophthalmology visual ability classification. We have included this in the method section on page 10 and lines 180 to 189 of the revised manuscript.
