# Supplementary material for: Willingness to pay for cataract surgery and associated factors among cataract patients in Outreach Site, North West Ethiopia
Source: PLoS One. 2021 Mar 24;16(3):e0248618. doi: 10.1371/journal.pone.0248618 (PMC7990211; doi:10.1371/journal.pone.0248618)
Supplement: Attachment — Submitted filename: Response to Reviewers.pdf [file pone.0248618.s011.pdf]

Response to Reviewers

Reviewer 2 comments and questions: -

Some of my previous comments have been addressed. However, there are still a few concerns.

1. The quality of the grammar and syntax is not of academic standard and may require the services of a professional to bring it up to the required standard.

   Author Response

   Dear reviewer thank you for your comments and questions. We have revised the English language of the manuscript by the professional language editor. Please see the whole revise manuscript.

2. The authors have explained their methods for calculating visual ability. However, they should include references for the calculation and examples of where this has been used in ophthalmic literature. Visual ability is not a term commonly used in ophthalmic literature and the thesis of this manuscript is the relationship between visual ability and willingness to pay.

   Author Response

Dear reviewer thank you for your comments and questions. The function of the visual system can be measured either objectively or subjectively. Visual acuity measurement is one of the objective indicators of the visual system functionality. However, it does not indicate the overall visual performance of an individual. Hence, we used the subjective measurement, the visual function/visual ability, to assess the overall visual performance. Hence it was measured by using adapted Visual Function 14 Index (VF-14) tool. Previously, numerous researchers used the tool as a standard. However, based on the socio-economic and cultural perspective of their study population, the tool was adapted. The response option was prepared with 5-point Likert's scale format. The scale ranged from the 0-4 value for the degree of visual difficulties 'unable to do'

(scale 0), ‘great difficulty’ (scale1), ‘moderate difficulty’ (scale 2), ‘little difficulty’ (scale 3) and ‘no difficulty’ (scale 4). Then each scale was multiplied by 25. So, the value of each response ranges from 0-100. After that, the factored amounts were summed up. Finally, VF index was computed by dividing the summed factored amounts to the number of checked boxes and the visual ability were categorized.

The calculation is elaborated across the studies, and the final classification is based on the International Council of Ophthalmology as normal or near-normal performance(≥50 visual ability score) and restricted performance(<50 visual ability score). We have included references in the revised manuscript at page

3. There are a few statements that are not factually correct. The opening sentence states that cataract is the second commonest cause of blindness worldwide; Cataract is the commonest cause of blindness globally. Refractive error is the commonest cause of visual impairment globally.

   Author Response

   Dear reviewer thank you for your comments and questions

4. Cataract surgical coverage and Cataract Surgical rate are two different indicators for assessing the quantity of cataract surgery

   Author Response

   Dear reviewer thank you for your comments and questions. We have revised the manuscript based o the raised issue. Please see page 4 from lines 51 to 53 of the revised manuscript.
