# Supplementary material for: Willingness to pay for cataract surgery and associated factors among cataract patients in Outreach Site, North West Ethiopia
Source: PLoS One. 2021 Mar 24;16(3):e0248618. doi: 10.1371/journal.pone.0248618 (PMC7990211; doi:10.1371/journal.pone.0248618)
Supplement: Attachment — Submitted filename: Comments_PONE-D-19-31270_reviewer.docx [file pone.0248618.s004.docx]

[PONE-D-19-31270]

**Manuscript Title:** Willingness to pay for cataract surgery and associated factors among cataract patients in outreach site, North West Ethiopia

I thank the editor for the opportunity to read this interesting work. Access to healthcare is a fundamental human right. Understanding the willingness to pay for cataract surgery and the associated factors are crucial in designing self-sustaining and efficient eyecare programs. I applaud the authors for contributing to the body of evidence.

However, as there was no line number given in the manuscript, sticky-notes were used (by the reviewer) to provide specific comments on the manuscript. It is highly recommended to use line numbers in the next versions. The sticky notes contain comments targeted to specific sentences or wordings; whereas the suggestions shared here are more general in nature. Authors are suggested to first go through the general-comments and then check the sticky-note-comments.

Suggestions for improvement are as follows:

1. The manuscript (including the abstract) demands massive improvement in writing. Moreover, there are typos and grammatical errors that need to be corrected.
2. WTP are health-state dependent, hence, it depends on the context and techniques applied (e.g. double bounded dichotomous choices) to elicit WTP. Sharing the tool (as supplementary documents) might be a good to option to let the reader understand the WTP estimation techniques applied in this study.
3. Generally outreach-sites are socio-demographically different from the general population. A comparison of socio-demographic characteristics between the sample and general population of North-West-Ethiopia or Ethiopia might provide a better understanding of the sample as-well-as understanding the differences in WTP in general context.
4. The ‘Introduction’ mentions about the mean costs of cataract surgery in Europe and USA. The cost of the surgery varies across the globe. As the study estimates the WTP for cataract surgery in Ethiopia, it would be wise to also state literatures that estimate WTP of other African and Asian countries (similar economies).
5. The ‘Introduction’ ends with a non-justifiable argument (Please check the sticky-note-comment). The manuscript states about the context-dependent WTP, however, it does not state any literature on context or health-state dependencies of estimated WTP. Also, please provide a detailed description of the context (i.e. WTP elicitation approach) in the method section.
6. There are various surgical techniques of cataract surgery (e.g. Small incision cataract surgery, phacoemulsification). For which technique of surgery respondents elicited their WTP for? Did they have full information or any prior knowledge on the surgical procedure(s)?
7. The ‘Study design and setting’ section should focus on the eyecare provided by the hospital. Please provide a summary of the eyecare facilities available in North West Ethiopia. It will help the reader to understand the representativeness of the setting (i.e. hospital) from which respondents were selected.
8. The ‘Study population and sampling technique’ section needs to improve its clarity in describing the sampling technique used for the study. Simple random sampling technique requires selecting subjects from a predetermined sampling frame. If the list of 1336 cataract patients was used as the sampling frame, then at least two things needs to be clarified:

(a) The sampling frame was prepared based on the patients visited the facility from June 10, 2018 to June 17, 2018; were the subjects chosen on a daily basis or based on an aggregated list.

(b) As the program provides free of cost cataract surgery, the subjects interviewed in this study should already have a preconceived estimation about the cost of the treatment; this has a definite impact on the elicited WTP (e.g. only 55 percent respondents elicited a non-zero WTP).

9. The description of bidding procedure given in page 5 needs clarity. How was initial bid (i.e. 1000 ETB) was chosen? A schematic representation of the DBDC experiment would help reader to visualize the context better. Please check the following reference:

[Islam M.N., Engles T., Hossain S., Sarker M., Rabbani A. “Willingness-to-pay for cataract surgeries among patients visiting eye-care facilities in Dhaka.” Applied Health Economics and Health Policy 17, no. 4 (2019): 545-554. DOI: 10.1007/s40258-019-00478-3]

10. About 45 percent respondents were not willing to pay a positive price for the service, how were these responses handled in the analysis? In addition, if some people were not willing to pay, were these people statistically different from those that were willing to pay?
11. The DBDC experiment ends with an interval of WTP. Did you check the interval regression techniques to estimate the associated factors? (If yes) Were the results different with an interval regression model?
12. The average WTP can be estimated using a constant only interval regression model. Although, 45 percent respondents did not elicit a positive WTP, their true WTP might be greater than zero but less than the last bid (750 ETB). In that case counting them as zero (as done in this manuscript) might not be pragmatic. Please check the following reference:

[Islam, M.N., Rabbani, A. & Sarker, M. “Health shock and preference instability: assessing health-state dependency of willingness-to-pay for corrective eyeglasses.” Health Economics Review 9, no. 1 (2019): 32. DOI:10.1186/s13561-019-0249-3]

13. The robustness of the Tobit models can be checked estimating coefficients of both censored and truncated samples as-well-as estimating the OLS coefficients. Please report the results (as supplementary documents). This practice will help the readers understand the robustness of the results obtained by the study.
14. The ‘Participant’s health-related characteristics’ section requires to provide sufficient details of the variables (what and how the measurements were taken; e.g. Systemic illness).
15. The *discussion* and *conclusion* need to be improved.
