# Supplementary material for: Willingness to pay for cataract surgery and associated factors among cataract patients in Outreach Site, North West Ethiopia
Source: PLoS One. 2021 Mar 24;16(3):e0248618. doi: 10.1371/journal.pone.0248618 (PMC7990211; doi:10.1371/journal.pone.0248618)
Supplement: Attachment — Submitted filename: Comments2_PONE-D-19-31270R1_Reviewer1.docx [file pone.0248618.s007.docx]

**[PONE-D-19-31270R1]**

**Title:** Willingness to pay for cataract surgery and associated factors among cataract patients in outreach site, North West Ethiopia

I thank the editor for the opportunity to review the manuscript once again. I also appreciate the authors’ for the efforts given to revise the manuscript according to the suggestions shared. However, there are a few important issues that require additional revisions or clarifications. The suggestions are as follows:

In reply to the authors’ response to the **Reviewer #1-Comment #1**:

The schematic description can provide a summarized picture of the bidding, however, it will not help the reader to replicate the experiment precisely in a similar manner. Including the tool as a supplementary document will provide the chance to the reader to understand what information was given to the respondents before and while eliciting WTP. Also, it would help the reader to design a similar questionnaire/tool for their experiments.

In reply to the authors’ response to the **Reviewer #1-Comment #5**:

In the previous review, the author was suggested to shed some light on the health-state dependency of the WTP and discuss how that would affect the results of their study. Looking at the available literature in this context would have been an appreciable initiative. However, the current version of the manuscript neither properly discussed nor provided any reference on their statements made in this context. There are researches that provide theoretical background as-well-as empirical evidences that WTP is health-state dependent. I would request the author to re-address comment #5.

In reply to the authors’ response to the **Reviewer #1-Comment #9**:

The schematic description of the DBDC experiment (shown on page 9) does not contain **the percentage of respondents on each of the decision nodes** and **the interval they ended up** before answering the maximum WTP and the **average maximum WTP** for different paths taken. It would provide the reader with a summary of the bidding experiment and would help understand whether the prices used for the experiment were reasonable or there were too many censored observations at any particular price points. I would strongly recommend the authors consider adding the information on the diagram shown on page 9.

Moreover, the statement made in lines 147-148 of page 8 needs a proper reference. Can you please add any literature that provides a background of this 35 percent out of pocket health expenditure as a proportion of the total healthcare spending of Ethiopia?

In reply to the authors’ response to the **Reviewer #1-Comment #12**:

The authors mentioned that 45 percent of the respondents who did not elicit any positive WTP were excluded from the further bidding experiments. There are three issues that need to be addressed:

1. The current schematic description (shown on page 9) does not include the initial question regarding the positive WTP. Please revise it accordingly.
2. If 45 percent of the respondents are not willing to pay any positive price, then, are there any systematic differences in their sociodemographic and/or health-related characteristics? Please provide both pooled and separated descriptive statistics in Table 1 and 2. Also, test the statistical significance of the differences by conducting either bivariate analyses or test a multivariable selection model for the two groups (i.e. =1 if positive WTP, 0 otherwise).
3. As these 45 percent of the respondents did not participate in the second phase of the bidding game, the Tobit model should also be estimated for both pooled and samples with only positive WTP. The authors have already estimated the models while addressing Comment #13. However, authors may consider showing and discussing them with the main results instead of just keeping in the appendix.

General suggestions:

The regression table shared as the supplementary document does not contain all the necessary information required to assess the robustness of the results. Please provide at least the standard errors and indications of statistical significance for all the models estimated.

There are a few minor grammatical errors and typos that require correction (e.g. see lines 21-23 on page 2). There are a few technical errors too that demand authors’ attention (e.g. lines 28-29 on page 2; **simple random sampling**).

If possible, please use an equation writing tool (such as equation writer in MS Word) and convert the lines 230-231 into an equation.

The data needs additional clarifications and missing the original variables. Standard practice should be to share the cleaned data according to the questionnaire/tools shared. Moreover, the MWTP data contains decimal characters; are these original MWTP or convert figures?
