# Supplementary material for: Willingness to pay for cataract surgery and associated factors among cataract patients in Outreach Site, North West Ethiopia
Source: PLoS One. 2021 Mar 24;16(3):e0248618. doi: 10.1371/journal.pone.0248618 (PMC7990211; doi:10.1371/journal.pone.0248618)
Supplement: Attachment — Submitted filename: Response to reviewers.docx [file pone.0248618.s008.docx]

**Response reviewers**

**Reviewer 1**

**Issues raised by the reviewer on comment #1**

**Authors’ response**

Dear reviewer, thanks for your comments and questions.

We have revised the manuscript as per the comment, and we have added the bidding approach tool we used as a supplementary file.

**Issues raised by the reviewer on comment #5**

**Authors’ response**

Dear reviewer, thanks for your comments and questions.

We have revised the main manuscript on page 5 from line 75 to 78. We have included the health state dependant nature of WTP.

**Issues raised by the reviewer on comment #9**

Dear reviewer, thanks for your comments and questions.

We have revised the main manuscript as per the given comment. We have analyzed the percentage of respondents at each node and stated in the main document on page 9.

We have also included the reference in the main manuscript for the issue raised on page 8.

**Issues raised by the reviewer on comment #12**

1. The current schematic description (shown on page 9) does not include the initial question regarding the positive WTP. Please revise it accordingly.

**Authors’ response**

Dear reviewer, thanks for your comments and questions.

We have revised the main manuscript accordingly, as per the comment on page 8.

2. If 45 percent of the respondents are not willing to pay any positive price, then are there any systematic differences in their sociodemographic and/or health-related characteristics? Please provide both pooled and separated descriptive statistics in Table 1 and 2. Also, test the statistical significance of the differences by conducting either bivariate analyses or test a multivariable selection model for the two groups (i.e. =1 if positive WTP, 0 otherwise).

**Authors’ response**

Dear reviewer, thanks for your comments and questions.

We have revised the main manuscript, and we present the pooled and separated descriptive statistics on table 1 and table 2. We have also run the logistic regression for WTP (1 = willing to pay and 0 = otherwise), and we have presented the finding in the main manuscript as table 3.

3. As these 45 percent of the respondents did not participate in the second phase of the bidding game, the Tobit model should also be estimated for both pooled and samples with only positive WTP. The authors have already estimated the models while addressing Comment #13. However, authors may consider showing and discussing them with the main results instead of just keeping in the appendix.

**Authors’ response**

Dear reviewer, thanks for your comments and questions.

We have revised the table and included in the main manuscript as table 5.

**For 1eneral suggestions raised by the reviewer**

1. **The regression table shared as the supplementary document does not contain all the necessary information required to assess the robustness of the results. Please provide at least the standard errors and indications of statistical significance for all the models estimated.**

**Authors’ response**

Dear reviewer, thanks for your comments and questions.

We have provided the standard error and p-value for the regression table and presented as table 5 in the main manuscript.

2. **There are a few minor grammatical errors and typos that require correction (e.g., see lines 21-23 on page 2). There are a few technical errors that demand authors’ attention (e.g., lines 28-29 on page 2; simple random sampling).**

**Authors’ response**

Dear reviewer, thanks for your comments and questions.

We have revised the manuscript as per the comment.

3. **If possible, please use an equation writing tool (such as equation writer in MS Word) and convert the lines 230-231 into an equation.**

**Authors’ response**

Dear reviewer, thanks for your comments and questions.

We have revised it and used the equation writing tool

4. **The data needs additional clarifications and missing the original variables. Standard practice should be to share the cleaned data according to the questionnaire/tools shared. Moreover, the MWTP data contains decimal characters; are this original MWTP or convert figures?**

**Authors’ response**

Dear reviewer, thanks for your comments and questions.

The MWTP is the converted one since initially, the MWTP was collected in Ethiopian Birr (ETB) and converted to USD for reporting and analysis. We have included the variables used for analysis in the main data, and the wealth index variable is computed from data collected from household assets by PCA.

**Reviewer 2**

**Question 1**

1. **Many of the references are out of date. There are more recent data on the prevalence and causes of global blindness and visual impairment. Flaxman et al 2017, Bourne et al 2017**

**Authors’ response**

Dear reviewer, thanks for your comments and questions.

We have revised the main manuscript as per the given comment.

2. **Several statements in the introduction are non- factual.
   E.g., Cataract accounts for a third of all global blindness, not 51%, as you stated.
   Universal Eye Health from the World Health Organization’s (WHO) Global Action Plan for eye health was launched in 2013, yet you provide references from 2001 and 2011.**

**Authors’ response**

Dear reviewer, thanks for your comments and questions.

We have revised the main manuscript as per the given comment on page 4, lines 49 to 51.

3. **Visual acuity, as classified by the WHO International classification of disease, is the most common descriptor for assessing vision. You insist on using a visual ability. The references you quote for visual ability are silent on this terminology.16, 18, 20-22, 24-27**

**Authors’ response**

Dear reviewer, thanks for your comments and questions.

We were considering to measure overall visual function with visual ability, which includes visual acuity as one component. As you stated, most literature used visual acuity to measure visual function. Visual acuity and visual ability have scope differences in which visual acuity is more specific, and visual ability is a general one. We want to acknowledge the comment you give us, and we appreciate the difference between visual ability and visual acuity. We use the references used for visual acuity because of both visual acuity, and visual ability reflects the visual function of the participants. We admit the comment you gave us, and if necessary, we can mention this in the limitation section.

4. **Incorrect grammar and syntax throughout the article.**

**Authors’ response**

Dear reviewer, thanks for your comments.

We tried to revise and edit the grammar of the main manuscript accordingly.

**Reviewer 3**

**Incorrect grammar and language**

**Authors’ response**

Dear reviewer, thanks for your comments.

We tried to revise and edit the grammar of the main manuscript accordingly.
