# Supplementary material for: Willingness to pay for cataract surgery and associated factors among cataract patients in Outreach Site, North West Ethiopia
Source: PLoS One. 2021 Mar 24;16(3):e0248618. doi: 10.1371/journal.pone.0248618 (PMC7990211; doi:10.1371/journal.pone.0248618)
Supplement: Attachment — Submitted filename: Comments3_PONE-D-19-31270R2_Reviewer1.docx [file pone.0248618.s009.docx]

**[PONE-D-19-31270R2]**

**Title:** Willingness to pay for cataract surgery and associated factors among cataract patients in outreach site, North West Ethiopia

I thank the editor for the opportunity to review the manuscript for the third time. I also appreciate the authors’ for the efforts given to revise the manuscript according to the suggestions shared. However, there are a few inconsistencies that need to be corrected. My comments are as follows:

1. Please mention the dates of data collection also in the main body.
2. The exchange rate used for the survey is the one of 2018. However, the world bank data the manuscript refers to in line no. 149 on page 8 is of the year 2009 (see reference no. 43). Moreover, the manuscript mentions that the out of pocket health expenditure is 35 percent of the total health expenditure in Ethiopia. Whereas, the report it refers to in reference no. 43 shows that it is 37 percent for Ethiopia. Authors should be very transparent and careful about the methodologies used and the methodologies described in the manuscript.
3. Line no. 152-154 mentions that the respondents were asked – “*Are you willing to pay some positive price for cataract surgery?*”, however, the questionnaire attached as a supplementary file states that, “*by considering the above scenario, if the service is vailing for you, are you willing to pay something for this service*”. Although, the sentences provide the same understanding of the question, there should not be any inconsistencies between what is written in the manuscript and what was actually asked. If the author wants to describe this as part of the methodology they applied; then, the exact question need not to be mentioned. This could be written differently, such as, “*After briefing the case scenario, all the participants were asked whether they are willing to pay any positive price for the proposed cataract surgery*”.
4. The intervals are not correctly specified in the schematic diagram. If anyone is not willing to pay 500 ETB, then the interval they fall into is 0 to <500 ETB. They intervals should be fixed by the experiment-design, not the average maximum WTP.
5. The dataset still does not contain the bidding experiment data. Sharing partial dataset will not help serving the right purpose of data-sharing.
